# Current evidence and therapeutic implication of PANoptosis in cancer

**DOI:** 10.7150/thno.91814

**Published:** 2024-01-01

**Authors:** Dickson Kofi Wiredu Ocansey, Fei Qian, Peipei Cai, Stephen Ocansey, Samuel Amoah, Yingchen Qian, Fei Mao

**Affiliations:** 1 Department of Laboratory Medicine, Lianyungang Clinical College, Jiangsu University, Lianyungang 222006, Jiangsu, P.R. China.; 2 Directorate of University Health Services, University of Cape Coast, Cape Coast CC0959347, Central Region, Ghana.; 3 The People's Hospital of Danyang, Affiliated Danyang Hospital of Nantong University, Zhenjiang 212300, Jiangsu, P.R. China.; 4 Department of Optometry and Vision Science, School of Allied Health Sciences, College of Health and Allied Sciences, University of Cape Coast, Cape Coast CC0959347, Central Region, Ghana.; 5 Department of Pathology, Nanjing Jiangning Hospital, Nanjing 211100, Jiangsu, P.R. China.

**Keywords:** PANoptosis, cancer, regulated cell death, therapy, immunity

## Abstract

Regulated cell death (RCD) is considered a critical pathway in cancer therapy, contributing to eliminating cancer cells and influencing treatment outcomes. The application of RCD in cancer treatment is marked by its potential in targeted therapy and immunotherapy. As a type of RCD, PANoptosis has emerged as a unique form of programmed cell death (PCD) characterized by features of pyroptosis, apoptosis, and necroptosis but cannot be fully explained by any of these pathways alone. It is regulated by a multi-protein complex called the PANoptosome. As a relatively new concept first described in 2019, PANoptosis has been shown to play a role in many diseases, including cancer, infection, and inflammation. This study reviews the application of PCD in cancer, particularly the emergence and implication of PANoptosis in developing therapeutic strategies for cancer. Studies have shown that the characterization of PANoptosis patterns in cancer can predict survival and response to immunotherapy and chemotherapy, highlighting the potential for PANoptosis to be used as a therapeutic target in cancer treatment. It also plays a role in limiting the spread of cancer cells. PANoptosis allows for the elimination of cancer cells by multiple cell death pathways and has the potential to address various challenges in cancer treatment, including drug resistance and immune evasion. Moreover, active investigation of the mechanisms and potential therapeutic agents that can induce PANoptosis in cancer cells is likely to yield effective cancer treatments and improve patient outcomes. Research on PANoptosis is still ongoing, but it is a rapidly evolving field with the potential to lead to new treatments for various diseases, including cancer.

## 1. Introduction

PANoptosis is a complex, multi-faceted agent of the immune response that has important implications for several diseases, including cancer. It is regulated by PANoptosomes, macromolecular complexes implicated in driving innate immune responses and inflammation 1,2. The assembly of PANoptosomes results in cell death that occurs through the collective activation of pyroptosis, apoptosis, and necroptosis 1. The precise molecular details of this process are unclear. Still, the presence of apoptosis and necroptosis markers in various body parts have been identified in several studies, leading to the hypothesis of possible PANoptosis in multiple diseases, including cancer 3,4. Studies that have analyzed the molecular changes of PANoptosis-related genes in cancer have reported distinct clusters that exhibit unique immune microenvironments, biological function, survival rate, and chemotherapy drug sensitivity 5-7. These findings highlight the therapeutic and prognostic value of PANoptosis, providing a new area for exploration. The PANoptosis pathway has also been studied in other diseases, such as inflammatory diseases, infections, and periodontitis 8. The study of PANoptosis also opens up a new frontier in RCD, not only in cancer but also in the context of stroke 4, neuroinflammation, and Alzheimer's Disease 9.

Despite some uncertainty about the specifics of the PANoptosis pathway, evidence suggests that it is deeply involved in cancer immunity and plays an important role in innate immune cells that detect and eliminate intracellular pathogens. Furthermore, several studies have shown that the expression profiles of key inflammatory cytokines are modulated in patients with cancer and that multiple cytokines are associated with disease severity, indicating that PANoptosis may be involved in regulating immune responses to cancer 10. While the exact mechanism by which PANoptosis drives immune responses and inflammation in cancer is poorly understood, it is believed to provide an "immune system workaround" to protect the host from cancer. According to a recent study, the sensors involved in PANoptosis may vary depending on the disease, but most infections will induce the formation of PANoptosomes, which unleash inflammatory cell death to protect the host 11. The therapeutic implications of PANoptosis in cancer are still emerging, but there is potential for the development of new treatments that target this pathway and improve existing therapies. For example, phosphorylated NFS1 was found to weaken oxaliplatin-based chemosensitivity of colorectal cancer (CRC) by preventing PANoptosis 12, implicating that the inhibition of NFS1 serves as a promising strategy for improving the outcome of platinum-based chemotherapy-induced PANoptosis in the treatment of CRC. Moreover, interferon regulatory factor 1 (IRF1) regulates PANoptosis to prevent CRC 13, and PANoptosis-based molecular clustering and prognostic signature predict patient survival and immune landscape in cancer 7. Thus, studies of the PANoptosis pathway and related molecules may inform the development of new therapeutic, diagnostic, and prognostic markers for cancer and provide a better understanding of the role of cell death in the regulation of immune responses to cancer.

The study reviews the role of regulated cell death (RCD) in cancer therapy, the interaction between these cell deaths, and the emergence and application of PANoptosis in treatments, particularly implications in developing therapeutic strategies for cancer. This will provide a timely overview of this new and promising field of research, highlighting a great deal of potential to develop new and effective PANoptosis-based cancer therapies.

## 2. Types of regulated cell death and application in cancer

**Figure 1:**
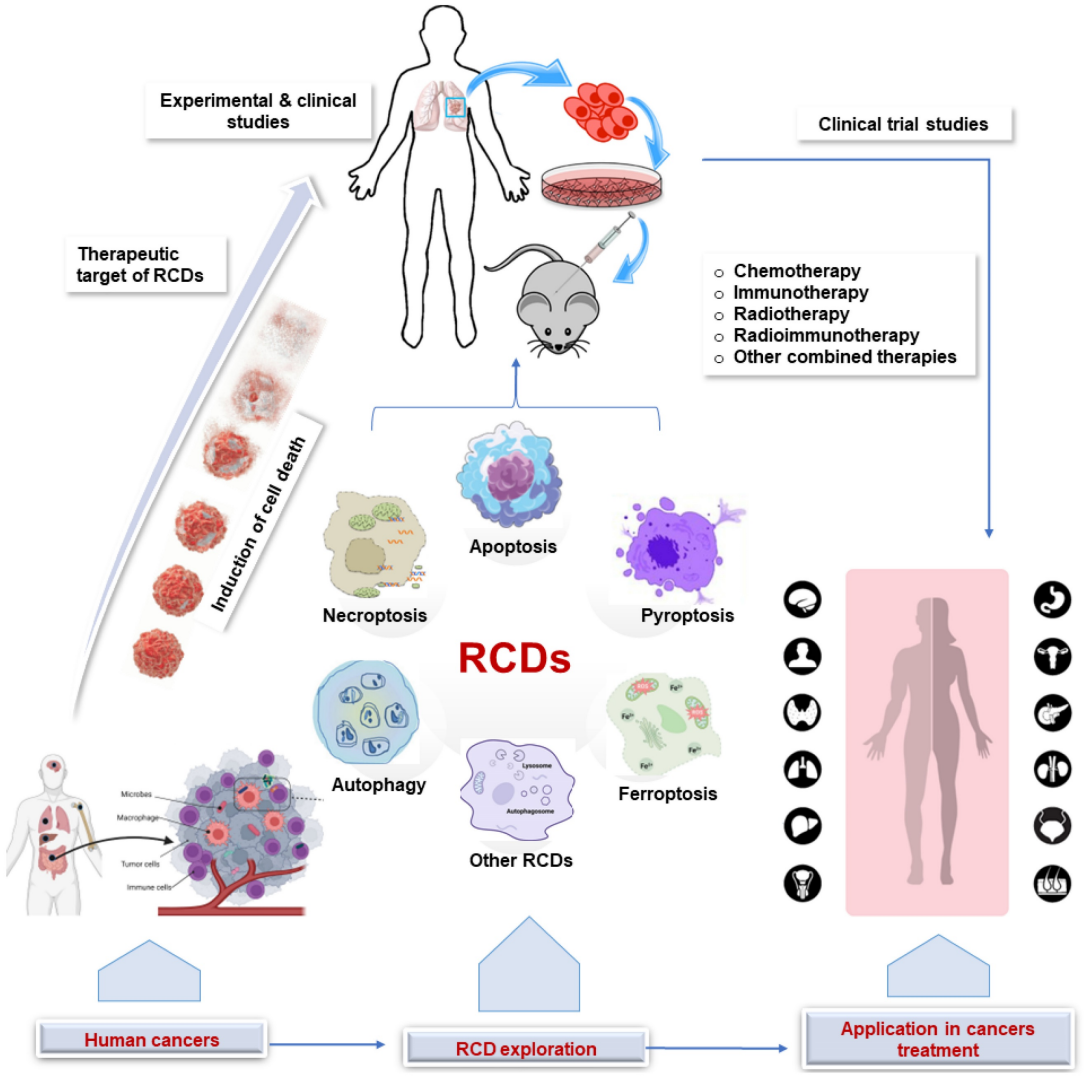
Application of RCD in cancer. Clinical and experimental studies continue to explore the target of RCDs in cancer. This sets the stage for clinical trials that ultimately lead to the application in human cancer treatment. RCD, regulated cell death.

Regulated cell death (RCD) is a broad term that encompasses all forms of cell death that are controlled by genes and occur through a series of biochemical steps, whereas programmed cell death (PCD) refers to the series of events that occur within a cell leading to its death 14,15. Thus, PCD is a subset of RCD and occurs during normal development and tissue homeostasis. Different types of RCD can be activated depending on the cell type and stimulus, each with distinct morphological and molecular characteristics, including apoptosis, autophagy, necroptosis, pyroptosis, ferroptosis, necrosis, and NETosis 16-18. In cancer therapy, RCD can be induced by a variety of treatments, including chemotherapy, radiation therapy, and immunotherapy. While chemotherapy drugs and radiotherapy work by interfering with the cell's ability to divide, leading to the death of cancer cells, immunotherapy is a relatively newer approach to cancer treatment in which the immune system is stimulated to attack cancer cells. The implications of the various RCDs in cancer therapy are summarized in Figure 1 and elaborated below.

### 2.1 Apoptosis

Apoptosis, the most well-known type of PCD, is a highly regulated process that is essential for normal development, tissue homeostasis, and eliminating unwanted or damaged cells 19. During apoptosis, the cell undergoes characteristic morphological changes, including cell shrinkage, nuclear fragmentation, chromatin condensation, and membrane blebbing. The cell's contents are then packaged into small membrane-bound vesicles called apoptotic bodies, which are engulfed and degraded by phagocytic cells without eliciting an inflammatory response 20,21. For over 30 years, a primary focus and goal of cancer treatment has been to develop therapies that help cancer cells die by apoptosis. In other words, researchers have intensely explored the clinical translation of apoptosis modulators into therapeutic approaches that target both the intrinsic and extrinsic apoptotic pathways. Among the strategies that target the intrinsic apoptotic pathway are selective BCL-2 inhibitors (Venetoclax, BCL201/S55746, APG-2575) 22-24, BCL-XL inhibitors (ABBV-155, A-1155463, WEHI-539, -1331852) 25,26, dual BCL-2 and BCL-XL inhibitors (APG-1252, Navitoclax, AZD4320, S44563 BM-1197) 27-29, MCL1 inhibitors (AMG 176, MIK665/S64315, AZD5991, A-1210477) 30-32, and inhibitor of apoptosis (IAP) proteins and SMAC mimetics (Birinapant/TL32711, LCL161) 26,33,34. The extrinsic apoptosis pathway is activated by signals from outside the cell that activate pro-apoptotic death receptors (DRs). DRs (including TNFR1, Fas (CD95, APO-1), DR3, DR4 (TRAILR1), DR5 (TRAILR2) and DR6) are transmembrane proteins that belong to the tumor necrosis factor receptor superfamily (TNFR) 35. Approaches that target the extrinsic apoptotic pathway to induce cancer death include the use of DR agonists such as Fas ligand, tumor necrosis factor-alpha (TNFα), TNF-related apoptosis-inducing ligand (TRAIL), Apo2L/TRAIL, and CD95L/FasL 26,36.

### 2.2 Necroptosis

Necroptosis is a form of programmed necrosis that occurs when the apoptotic pathway is inhibited. Necroptosis is characterized by programmed mitochondrial dysfunction and plasma membrane rupture induced by various stimuli, including TNF-alpha, Toll-like receptor (TLR) ligands, and chemotherapeutic drugs 37. Unlike apoptosis, necroptosis involves the release of cellular contents into the extracellular space, which can trigger an inflammatory response. Necroptosis is regulated by several essential proteins, including receptor-interacting protein kinases (RIPKs) and mixed lineage kinase domain-like pseudokinase (MLKL) 38,39. Necroptosis can have both tumor-promoting and tumor-suppressing effects in cancer 40. On the one hand, necroptosis can trigger an inflammatory response that can recruit immune cells to the tumor and promote its clearance. On the other hand, necroptosis can also promote tumor metastasis by helping cancer cells detach from the primary tumor and invade other tissues 40,41. Some of the specific applications of necroptosis in cancer therapy include necroptosis-inducing drugs, necroptosis inhibitors (receptor-interacting serine/threonine-protein kinase (RIPK)1 inhibitors, RIPK3 inhibitors, MLKL inhibitors), and necrotic cell debris removal. Drugs that induce necroptosis in cancer cells are still in the early stages of development, but they have shown promise in preclinical studies. For example, drugs such as Neoalbaconol 42, Matrine 43, Erigeron breviscapus 44, and inorganic salts and metal complexes (CoCl2, LiCl, Cisplatin, Rhenium(V) oxo complexes) 45-47 have been shown to induce necroptosis in a variety of cancer cell lines and to suppress tumor growth in animal models.

### 2.3 Pyroptosis

Pyroptosis is characterized by the release of proinflammatory cytokines, including IL-1β and IL-18, and the formation of large membrane pores 48. It is a caspase-independent form of cell death triggered by stimuli such as microbial pathogens, damage-associated molecular patterns (DAMPs), and certain chemotherapeutic drugs. It can also occur spontaneously in cancer cells that have mutations in certain genes, such as caspase (CASP)1, CASP3, and NOD-like receptor protein 3 (NLRP3) 49,50. Pyroptosis can have both tumor-promoting and tumor-suppressing effects in cancer. On the one hand, pyroptosis can trigger an inflammatory response that can recruit immune cells to the tumor and promote its clearance. On the other hand, pyroptosis can also promote tumor metastasis by helping cancer cells detach from the primary tumor and invade other tissues 51. Studies show that Gasdermins (GSDMs)-driver pyroptosis also participates in the inflammatory cell death, PANoptosis, which makes a significant sense to the initiation and progression of diseases 52, including tumors, where *GSDM genes* are highly upregulated and associated with tumor microenvironment (TME) features, prognosis, clinical characteristics, and stemness scores in several cancer types 53. Thus, pyroptosis is a promising target for cancer therapy. The association of *GSDM genes* (and their regulation of PANoptosis) with tumor behaviors and probable participation in carcinogenesis presents it as a potential therapeutic target in pan-cancer. Several studies have explored pyroptosis as a new bridge in tumor immunity, targeting GSDM and related pathways 54,55. In summary, targets for pyroptosis in cancer include pyroptosis-inducing drugs (shikonin, ferrostatin-1, cisplatin), pyroptosis inhibitors (necrostatin-1, MCC950), and pyroptotic cell debris removal (A11) 56,57.

### 2.4 Autophagy

Autophagy is a cellular recycling process that is critical in maintaining cellular homeostasis by degrading and recycling cytoplasmic contents, including organelles and proteins. During autophagy, the cell forms a double-membraned autophagosome that sequesters the cellular components to be degraded and fuses with a lysosome to form an autolysosome. Lysosomal enzymes then degrade the contents, and the resulting molecules are released back into the cytosol for reuse 58,59. Hyper-activation of autophagy is also believed to cause autophagy-dependent cell death (ADCD), a caspase-independent form of programmed cell death. ADCD serves as a type of cellular demise that may act as a backup cell death program in apoptosis-deficient tumors, presenting a potential cancer cell death target 60. Researchers are developing therapies that target the autophagy pathway, specifically in cancer cells, including the inhibition of autophagy-related proteins that are essential for tumor growth and metastasis 61. In cancer research, studies have explored autophagy-inducing drugs such as Vorinostat and Hydroxychloroquine 62, autophagy inhibitors such as 3-Methyladenine and Chloroquine 63,64, and autophagy pathway-specific drugs like ATG4B 65.

### 2.5 Ferroptosis

Ferroptosis is a regulated form of cell death that depends on iron and lipid peroxidation. It is characterized by the accumulation of lipid peroxides and the absence of caspase activation, distinguishing it from apoptosis and necrosis 66. Ferroptosis is initiated by the depletion of glutathione or the inhibition of glutathione peroxidase 4, leading to lipid peroxidation and membrane damage 67,68. Studies have implicated ferroptosis in a variety of physiological and pathological processes, including cancer, where it plays a role in tumor suppression and drug resistance and presents promising therapeutic potential 69. In other words, ferroptosis plays an essential role in preventing cancer development but is often suppressed in many types of cancer, suggesting that regulating ferroptosis could be a promising way to treat cancer 70. Several substances are under investigation for their ability to regulate ferroptosis to reduce tumor progression, including Simvastatin 71, P53 72, and BAP1 70. A study found that KRAS mutant in tumors is associated with suppressed ferroptosis and associated gene expression changes that reduce adverse outcomes 73, presenting a novel potential avenue for therapeutic approaches in cancer. In other studies, Glutathione S-transferase zeta 1 (GSTZ1), an enzyme in the catabolism of phenylalanine, enhances sorafenib-induced ferroptosis by inhibiting the NRF2/GPX4 axis 74. Interestingly, although many ferroptosis regulators such as GPX4, SLC7A11, and FTH1 are obviously increased in certain cancers compared with normal tissues, the treatments targeting these regulators might vary in effectiveness across different cancers 70.

### 2.6 Other types of regulated cell death

Other forms of regulated cell death include NETosis, entosis, parthanatos, and mitotic catastrophe. Moreover, immunogenic cell death (ICD), lysosome-dependent cell death (LDCD), alkaliptosis, and oxeiptosis have also been documented as emerging RCD pathways 75,76. NETosis, or neutrophil extracellular trap formation, occurs in neutrophils in response to infection or inflammation. In the tumor microenvironment, NETosis can play both a tumor-suppressive role by trapping and killing cancer cells and a tumor-promoting role by providing a scaffold for tumor cell attachment and migration and releasing pro-angiogenic and pro-inflammatory factors that promote tumor growth 77,78. NETosis is distinct from apoptosis and necrosis and is characterized by the release of DNA, histones, and antimicrobial peptides from neutrophils and the formation of extracellular traps 79. On the other hand, entosis occurs when one living cell invades and engulfs another living cell's cytoplasm. This process involves the formation of cell-in-cell structures, where the engulfed cell is degraded and eventually dies 80. Entotic cell death occurs in a non-apoptotic manner and usually involves the lipidation of microtubule-associated protein light chain 3 (LC3) onto entotic vacuoles. The process of entosis is triggered by the loss of attachment to the extracellular matrix and occurs in response to several stimuli, such as hypoxia, nutrient starvation, and chemotherapy 81. The role of entosis in cancer is complex and multifaceted. Entosis can lead to the death of cancer cells, but it can also promote tumor growth and metastasis 82. This suggests that entosis could be a potential target for therapeutic intervention, and several entosis-targeting therapies are currently in development 83.

Parthanatos is triggered by excessive activation of poly(ADP-ribose) polymerase-1 (PARP-1) and the subsequent release of apoptosis-inducing factor (AIF). Parthanatos can occur independently of caspases and is involved in the pathogenesis of various cancers and neurodegenerative diseases 39,84. A number of parthanatos-targeting therapies are currently in preclinical or clinical development. For example, some studies have shown that inhibiting PARP1, a key enzyme involved in parthanatos, can suppress tumor growth in animal models 85,86. Other studies have shown that stimulating parthanatos with certain drugs can kill cancer cells in vitro and in vivo 86,87. Mitotic catastrophe is another type of PCD that occurs in response to errors in cell division, leading to abnormal mitotic events and genomic instability. During mitotic catastrophe, cells undergo premature or delayed mitosis, leading to chromosomal instability and the activation of the DNA damage response. If the DNA damage response is not resolved, cells will ultimately undergo PCD 88. Mitotic catastrophe can be triggered by a variety of factors, such as chemotherapeutic drugs, radiation, and viral infection 88. Mitotic catastrophe plays a vital role in cancer prevention and suppression. Mitotic failure can lead to senescent cells, which could suppress tumor growth by blocking the proliferation of neighboring cancer cells and recruiting immune cells to the tumor microenvironment 89. Notwithstanding, cancer cells can often evade mitotic catastrophe and senescence, which allows them to continue to proliferate and form tumors 90. Chemotherapeutic agents such as vinca alkaloids and taxanes can trigger mitotic catastrophe. Such agents can also cause cancer cells to undergo immunogenic cell death (ICD), which allows therapeutic responses to last beyond treatment discontinuation due to the induction of an antitumor immune response 91. Researchers are also investigating the potential of combining mitotic catastrophe-inducing therapies with other cancer therapies, such as chemotherapy and immunotherapy. The goal of these combination therapies is to make cancer cells more sensitive to treatment and to prevent the development of drug resistance 92,93. In one such study, 212Pb-radioimmunotherapy was found to potentiate paclitaxel-induced cell-killing efficacy by perturbing the mitotic spindle checkpoint, causing increased mitotic catastrophe and cell death 92.

ICD, defined by chronic exposure to DAMPs in the TME, stimulates the dysfunctional antitumor immune system and contributes to long‐lasting protective antitumor immunity. ICD induction has emerged as a novel cancer therapeutic approach. Some of the reported ICD inducers include bleomycin (an antitumor antibiotic glycopeptide), cyclophosphamide, cardiac glycosides (digoxin, digitoxin, ouabain, and lanatoside C), shikonin, anthracyclines (doxorubicin, idarubicin, and mitoxantrone), and hypericin photodynamic therapy (Hyp-PDT) 94. For example, anthracyclines induce ICD in human prostate cancer, ovarian cancer, and acute lymphoblastic leukemia cells by increasing the maturation of DCs and tumor cell uptake by DCs, stimulating tumor-specific IFN-γ-producing T cells, reducing Tregs 95. In LDCD, the lysosome serves as an anti-cancer target, where lysosome-disrupting agents trigger lysosomal membrane permeabilization (LMP), cathepsin protease release, and subsequent LDCD. Depletion of cancer cells through LMP is an attractive therapeutic strategy, holding particular promise for combating apoptosis-resistant cancer cell populations. Several lysosome-disrupting agents (lysosomotropic agents, sphingolipids, nanoparticles) are currently under investigation or in clinical development for cancer and other indications 96. Alkaliptosis, a pH-dependent form of RCD, has also been recently identified as a new strategy for cancer therapy across multiple tumor types. For example, a G protein-coupled receptors (GPCRs)-targeted small molecule called JTC801 has been demonstrated to suppressed tumor growth in xenograft, orthotropic, lung metastases, and genetically engineered mouse models of pancreatic cancer in vivo by inducing alkaliptosis, but not apoptosis, necroptosis, ferroptosis, and autophagy-dependent cell death 97. Moreover, oxeiptosis is recently identified as a reactive oxygen species (ROS)-sensitive, caspase-independent, non-inflammatory RCD pathway. Data indicates that targeting oxeiptosis-mediated tumor suppression presents a novel approach to treating cancers 98. Regardless, data on these cell death pathways are largely lacking and calls for further exploration in future studies.

## 3. Key molecular interaction between the types of programmed cell death

**Figure 2:**
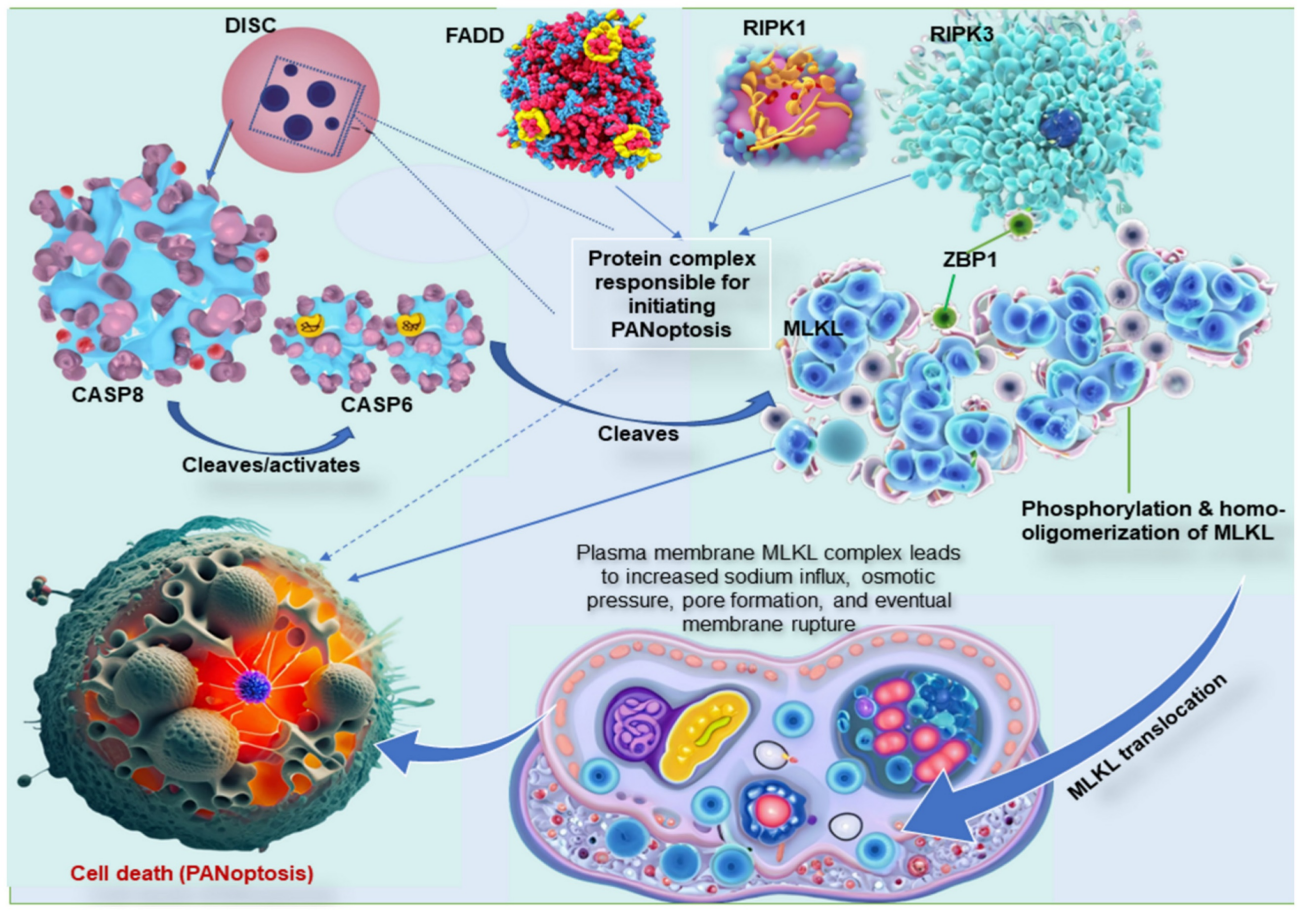
Key molecular interaction between the types of programmed cell death. ZBP1 is essential for forming the PANoptosome, a protein complex that causes cell death. ZBP1 does this by recruiting RIPK3, another protein in the PANoptosome. RIPK3 then phosphorylates MLKL, a third protein in the PANoptosome. This phosphorylation causes MLKL to move to the plasma membrane, forming pores that kill the cell. In other words, ZBP1 is the glue that holds the PANoptosome together. It recruits the other complex proteins and helps them function properly. MLKL must form clumps (oligomerize) to move to the plasma membrane, forming pores that kill the cell. This movement to the plasma membrane happens before cell death. MLKL, either by itself or with the help of other proteins, causes sodium to flow into the cell. This influx of sodium increases the pressure inside the cell, which eventually causes the cell membrane to burst. DISC, comprising of Fas, FADD, and caspase-8, is a multi-protein complex that initiates PANoptosis, whereas RIPK1 also complexes with RIPK3 and FADD to initiate PANoptosis. ZBP1, Z-DNA binding protein 1; RIPK, receptor interacting serine/threonine kinase; MLKL, mixed lineage kinase domain like pseudokinase; DISC, death-inducing signaling complex; FADD, Fas-associated death domain.

The three common pathways, necroptosis, pyroptosis, and apoptosis, have unique mechanisms, proteins, and byproducts. The key molecular interactions between these cell death pathways are complex and involve a number of different proteins 99. The most important interactions include CASP8, RIPK1, RIPK3, MLKL, and Z-DNA binding protein 1 (ZBP1). CASP8 is a central player in all three PCD pathways 100. In apoptosis, CASP8 is activated by a protein complex called the death-inducing signaling complex (DISC). The DISC also contains the Fas-associated death domain (FADD) proteins and RIPK1. Once CASP8 is activated, it cleaves and inactivates RIPK1, preventing the formation of the necrosome and necroptosis 99,101. RIPK1 is another crucial player in all three PCD pathways. In apoptosis, RIPK1 is inactivated by CASP8. However, if CASP8 is inhibited, RIPK1 can form a complex with RIPK3 and FADD called the necrosome, a complex responsible for initiating necroptosis 102. In the interaction between these cell death pathways, RIPK3 is recruited to the necrosome by RIPK1 and phosphorylates MLKL, which is the final effector of necroptosis. Once RIPK3 phosphorylates MLKL protein, it is translocated to the plasma membrane and forms pores that lead to cell death 84,99. Moreover, ZBP1, an essential protein in these mechanisms, interacts with both CASP8 and RIPK3 and is required to assemble the PANoptosome (Figure 2) 103. In addition to these key molecular interactions, there are a number of other proteins that are involved in regulating pyroptosis, apoptosis, and necroptosis. These proteins include inflammasomes, caspases, kinases, and phosphatases 15,17,35,104.

The complex interplay between these proteins allows the cell to fine-tune its response to various stimuli and choose the appropriate PCD pathway. For example, if the cell is infected with a virus, it may undergo pyroptosis to release inflammatory cytokines and alert the immune system. However, if the cell is damaged beyond repair, it may undergo apoptosis or necroptosis to prevent the spread of damage to other cells 15,17,35,104. Studies have also shown that dysregulated PCD can lead to abnormal growth of cells, a hallmark feature of many cancer pathologies, causing tumor growth. An integral protein, ZBP1 participates in initiating the NLRP3 inflammasome, a known facilitator of pyroptosis. It is also demonstrated to bind to RIPK3, a regulator of necroptosis. Moreover, a unique binding partner to ZBP1, adenosine deaminase acting on RNA 1 (ADAR1), is involved in RNA editing, stress mechanisms, and diseases, including cancers 105. Research on the molecular interactions between PCDs is still ongoing and rapidly evolving and harbors the potential to lead to new treatments for diseases like cancer.

## 4. The emergence of PANoptosis

The term "PANoptosis" was first coined in 2019 by a team of researchers led by Subbarao Malireddi 106. In their study, the researchers showed that a protein complex, later dubbed PANoptosome, could induce a unique type of cell death characterized by features of pyroptosis, apoptosis, and necroptosis 106,107. Prior to the discovery of PANoptosis, researchers had observed that certain types of cell death shared components of multiple PCD pathways. However, the molecular mechanisms underlying these hybrid cell death processes were poorly understood. The discovery of the PANoptosome and its role in PANoptosis provided a new framework for understanding these hybrid cell death processes 2,103,108. After the discovery and description of "PANoptosis" in 2019, other key milestones in the historical evolution of PANoptosis include the demonstration of the role of PANoptosis in the development of sepsis 107,109 and tumors 13,110. Moreover, in 2022, researchers show that PANoptosis plays a role in the neurodegeneration that occurs in Alzheimer's disease 9.

Therefore, since 2019, there has been a rapid increase in the number of studies published on PANoptosis. These studies have shed light on the molecular mechanisms of PANoptosis and its role in various diseases 6,10,52,111,112. PANoptosis is now thought to be a critical defense mechanism against infection, but it is also believed to play a role in the development of a variety of diseases, including cancer, sepsis, inflammatory bowel disease, and Alzheimer's disease 99. In summary, the historical evolution of the term PANoptosis is, therefore, relatively short, but it is a rapidly evolving field of research with the potential to lead to new treatments for various diseases.

## 5. Evidence of PANoptosis in cancer

PANoptosis serves as a multifaceted agent of the immune response with implications in driving innate immune responses and inflammation and playing a crucial role in cancer. Recent studies have demonstrated the participation of PANoptosis in several cancers. The evidence from comprehensive analyses, molecular clustering studies, and experimental validations in specific cancer cell lines collectively support the involvement of PANoptosis in cancer, highlighting its potential as a target for therapeutic interventions. For example, PANoptosis genes were found to contribute to the tumor microenvironment in lower-grade glioma patients 113, while PANoptosis-based molecular clustering demonstrated predictive capabilities for the survival and immune status of colon cancer patients 7, providing evidence of its involvement in cancer biology. By analyzing 1203 glioma samples from three public databases, the researchers found that PANoptosis is involved in tumor microenvironment interaction and patients' prognosis, and distinct PANclusters have their own molecular, immunological, and clinical profiles 113. In a similar report, a study identified three distinct PANoptosis patterns in 1316 gastric cancer patients, each PANoptosis pattern has its own molecular, clinical, and pathological characteristics 3. Characterizing the PANoptosis patterns predicts immunotherapy response and survival, providing a roadmap for patient stratification, not only in gastric cancer but also across pan-cancer 3. In a proof-of-concept study, PANoptosis was shown to cause cell death in melanoma cancer cell lines. This experimental evidence reinforces the role of PANoptosis in cancer, specifically in inducing cell death 114. Other authors indicate that PANoptosis genes are aberrantly expressed in most cancer types and are consistent with the validation of PYCARD expression, a PANoptosis gene. PANoptosis gene expression and score correlate significantly with patient survival in 21 and 14 different cancers, respectively 6, and contribute to predicting immunotherapy response in patients with tumors. A recent study found that genes and gene clusters involved in PANoptosis are associated with patient survival, immune system function, and cancer-related biological processes and pathways. The study also found that a calculated risk score can be used to predict which patients have a worse prognosis. The risk score is associated with the abundance of immune cells, the cancer stem cell index, checkpoint expression, and the response to immunotherapy and chemotherapy drugs 7. Thus, PANoptosis-based molecular clustering and prognostic signatures can be used to predict patient survival and tumor microenvironment characteristics.

There is evidence of mutations in PANoptosis-related genes, among which *NLRP3* had the highest mutation frequency, and *ZBP1, AIM2*(absent in melanoma 2)*, NLRP3,* and *GSDMD* (Gasdermin D) had the highest copy number variation. In tumor samples, *NLRP3, CASP7, RIP1* (receptor-interacting protein 1), and *RIP3* are downregulated 7. In another study, researchers identified a prognostic signature based on the expression of genes involved in PANoptosis. The signature was found to predict overall survival in patients with cutaneous melanoma, and it also provided insights into the immune infiltration landscape of the tumor 115. The prognosis-associated PANoptosis-related genes (*ZBP1, MAP3K7,* and *RBCK1*) were highly elevated in the low-risk group and less expressed in the high-risk group, suggesting the participation of PANoptosis in cancer progression and as a potential target for therapeutic intervention in cancers 115. Long non-coding RNAs (lncRNAs) are crucial modulators of cancer occurrence and progress. Researchers have identified several lncRNAs associated with PANoptosis and metastasis in colon adenocarcinoma. While the expression of MIR22HG and SFTA1P are decreased in colon adenocarcinoma compared with healthy individuals, SNHG6, FAM222A-AS1, SNHG11, RHPN1-AS1, SNHG16, SNHG1, and SNHG7 are highly expressed in tumors versus normal tissues. Further analysis indicated that the expression level of lncRNA SNHG7 correlates with tumor stage and lymph node metastasis. It also contributes to drug resistance and significantly relates to prognosis 111. In addition, the PANoptosome has been identified as a cytoplasmic multimeric protein complex that controls PANoptosis 116. This complex can engage three key modes of PCD, pyroptosis, apoptosis, and necroptosis 117, all of which are implicated in cancer. Cytosolic innate immune regulators and sensors, such as ZBP1, RIPK1, and AIM2promote the assembly of PANoptosomes to drive PANoptosis 2, and studies highlight recent progress in cancer treatment based on PANoptosis and ferroptosis induced by other small-molecule compounds, immune checkpoint inhibitors, and nanoparticles 104. Some of the studies that report the involvement of PANoptosis in cancer are summarized in Table 1.

## 6. PANoptosis in immunity and its associated molecular mechanisms in cancer

**Figure 3:**
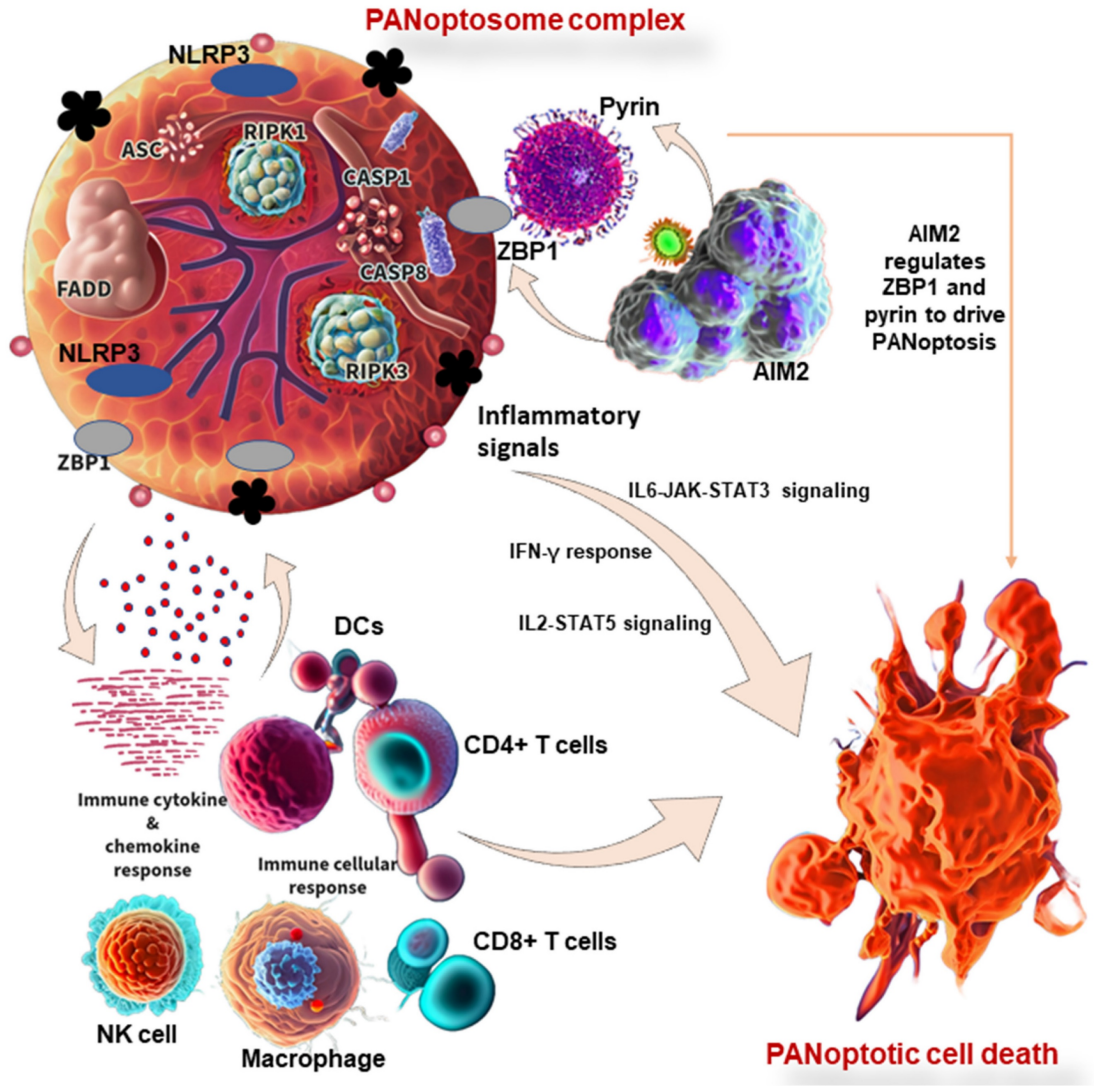
PANoptosis in immunity and its associated molecular mechanisms in cancer. PANoptosome complex consists of ZBP1 and NLRP3 as putative sensors, ASC and FADD as adaptors, and RIPK1, RIPK3, CASP1, and CASP8 as catalytic effectors. AIM2 regulates ZBP1 and pyrin to drive inflammatory signaling and PANoptosis. In other words, AIM2, ZBP1, and pyrin are members of a large multi-protein complex along with CASP1, CASP8, ASC, RIPK3, RIPK1, and FADD, that promote PANoptosis. PANoptosis drives innate immune responses and inflammation, influencing immune cells, cytokines, and chemokines. ZBP1, Z-DNA binding protein 1; AIM2, absent in melanoma 2; CASP, caspase; NLRP1, NLR family pyrin domain containing 1; FADD, Fas-associated death domain; ASC, apoptosis-associated speck-like protein containing a caspase recruitment domain; RIPK, receptor interacting serine/threonine kinase; IL, interleukin; NK, natural killer, DCs, dendritic cells; JAK, Janus kinase; STAT, signal transducer and activator of transcription; IFN-γ, interferon-gamma.

The innate immune system is made up of barriers that aim to keep pathogens and cancer cells out of the body or limit their ability to spread. During invasion, the innate immune system recognizes the tumor cell or conserved microbial molecules and engages a rapid response by producing inflammatory mediators and activating PCD pathways, including PANoptosis, pyroptosis, apoptosis, and necroptosis 112. Studies have shown that PANoptosis is implicated in driving innate immune responses and inflammation, and it cannot be individually accounted for by pyroptosis, apoptosis, or necroptosis alone 8,112. ZBP1 has been identified as the first innate immune sensor to form a PANoptosome and induce PANoptosis. It is reported that AIM2 regulates the innate immune sensors ZBP1 and pyrin to drive inflammatory signaling and PANoptosis, providing host protection. AIM2, ZBP1, and pyrin are members of a large multi-protein complex along with CASP1, CASP8, ASC, RIPK3, RIPK1, and FADD, that promotes inflammatory cell death (PANoptosis) (Figure 3) 11. Moreover, pathogens induce inflammatory PCD in the form of pyroptosis, apoptosis, and necroptosis (PANoptosis), where the Zα2 domain of ZBP1 is required to promote the activation of inflammasome/PANoptosis 109. CASP6 facilitates the RIP homotypic interaction motif (RHIM)-dependent binding of RIPK3 to ZBP1, contributing to ZBP1-mediated inflammasome activation, PANoptotic cell death, and host defense 119. Therefore, studies have reported the functional significance of CASP6 in PANoptosis and its impact on cancer development, indicating a potential therapeutic target and strategy for tumor treatment 108. In addition to cancer, the implications of PANoptosis in infectious diseases and autoimmunity and its potential as a therapeutic target have been the focus of ongoing research 1,8.

In cancer studies, comprehensive pathway analysis shows that PANoptosis score positively correlates with several pathways associated with immune and inflammatory responses in pan-cancer, including IL6-JAK-STAT3 signaling, the interferon-gamma (IFN-γ) response, and IL2-STAT5 signaling. Moreover, PANoptosis score is significantly associated with the tumor microenvironment, immune-related genes, and the infiltration levels of most immune cells, including NK cells, CD4^+^ T cells, CD8^+^ T cells, and DCs 6. These observations provide PANoptosis components in cancer as potential prognostic and therapy biomarkers. Some of the innate immune cells that have been shown to be involved in PANoptosis are discussed below.

### 6.1 Macrophages

Macrophages are large phagocytic cells that play a crucial role in innate immunity by recognizing and engulfing pathogens, foreign substances, and cell debris. They are involved in the induction of PANoptosis in some studies. For example, a study found that PANscore, a risk-scoring system based on the PANoptosis patterns, is significantly associated with dense infiltration of M2 macrophage and cancer-associated fibroblasts, high expression of TGF-β, and low expression of immune checkpoints 3. Infection of macrophages with pathogens results in robust cell death and the hallmarks of PANoptosis activation. Combined deletion of the PANoptotic components CASP1 (CASP1), CASP8, CASP11, and RIPK3 essentially protects macrophages from cell death induced by pathogens, while deletion of individual components provides reduced or no protection. Further analysis indicates that molecules from the apoptotic, pyroptotic, and necroptotic cell death pathways interact to form a single molecular complex termed the PANoptosome 107.

Another study demonstrated that CASP6 mediates innate immunity, inflammasome activation, and activation of PANoptosis, partly by promoting the differentiation of alternatively activated macrophages (M2) 119. These observations underscore the involvement of macrophages in the activation of PANoptosis and the formation of a PANoptosome complex.

### 6.2 Dendritic cells (DCs)

**Figure 4:**
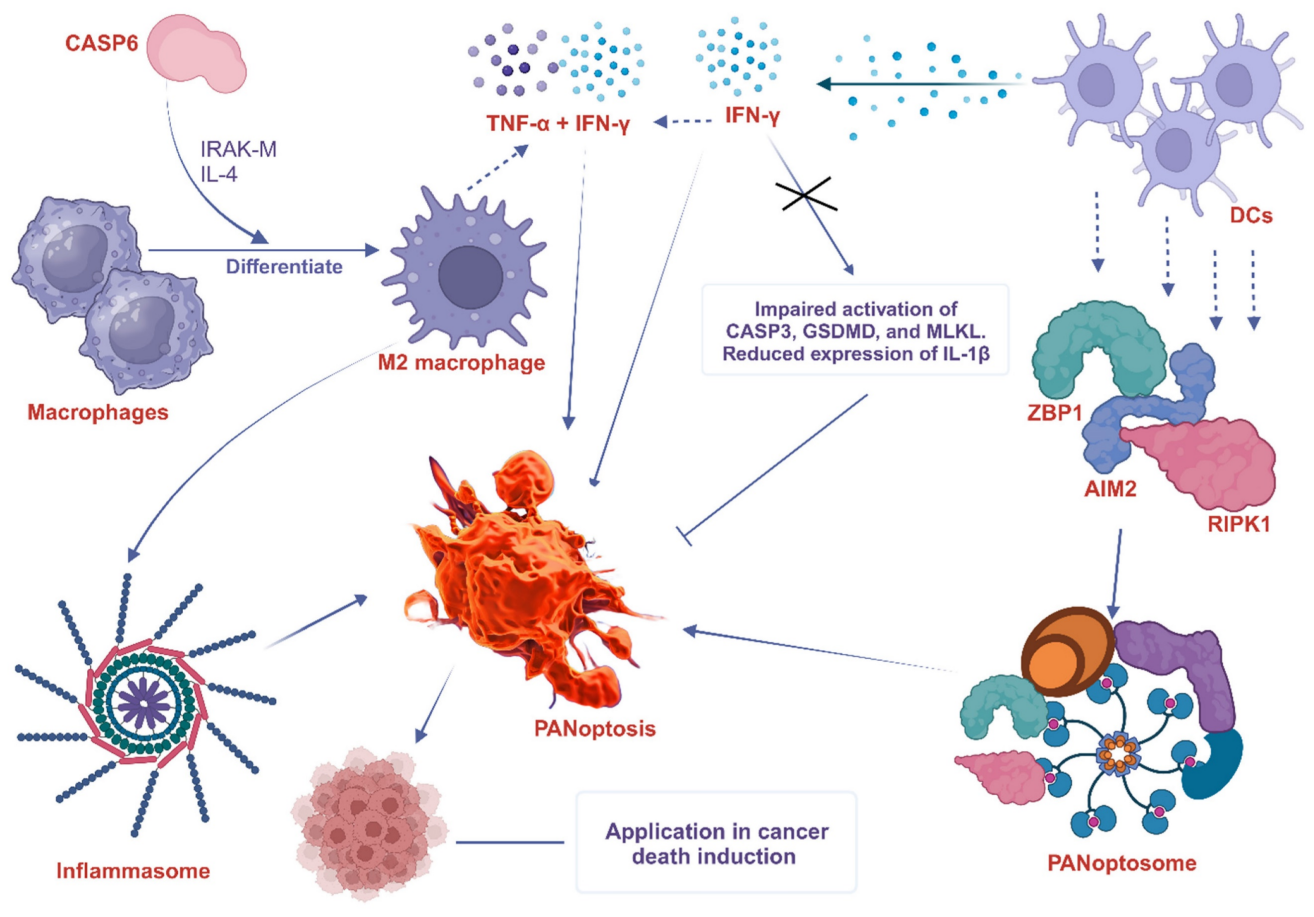
The involvement of macrophage and DC in PANoptosis. CASP6 plays a key role in the innate immune system, inflammasome activation, and PANoptosis. It does this partly by promoting the differentiation of macrophages into M2 macrophages. CASP6 also plays an essential role in activating macrophages by cleaving IRAK-M, and exposure to IL-4 also enhances M2 activation, further increasing the expression of CASP6. DCs express cytosolic innate immune sensors and regulators such as ZBP1, AIM2, and RIPK1. These proteins help to assemble the PANoptosome, which triggers PANoptosis. IFN-γ produced by DCs promotes PANoptosis in mice. IFN-γ deficiency impairs the activation of PANoptosis-specific markers such as CASP3, GSDMD, and MLKL and reduces the expression of IL-1β. The combined effects of TNF-α and IFN-γ also drive PANoptosis. ZBP1, Z-DNA binding protein 1; AIM2, absent in melanoma 2; CASP, caspase; RIPK, receptor interacting serine/threonine kinase; MLKL, mixed lineage kinase domain like pseudokinase; IFN-γ, interferon-gamma; IL, interleukin; TNF-α, tumor necrosis factor alpha; DCs, dendritic cells; IRAK-M, interleukin-1 receptor-associated kinase-M; GSDMD, Gasdermin D.

DCs are specialized antigen-presenting cells that play a crucial role in initiating adaptive immune responses. Although there is no direct evidence of the interaction between DCs and PANoptosis, they are believed to be involved in the induction and regulation of PANoptosis. A study found that the PANoptosis score significantly correlates with the infiltration levels of most immune cells such as DCs, as well as the tumor microenvironment and immune-related genes 6. Some potential ways in which DCs can participate in PANoptosis include cytokine production, expression of death receptors, and formation of the PANoptosome. For instance, DCs are known to trigger the expression of cytosolic innate immune sensors and regulators such as ZBP1 120, AIM2 121, and RIPK1 122, which promote the assembly of PANoptosomes to drive PANoptosis. Interestingly, the expression of ZBP1 is significantly elevated in not only DCs but also neutrophils, macrophages, natural killer (NK) cells, basophils, CD4^+^ T cells, and regulatory T (Treg) cells of infected patients compared with healthy controls 120. Again, interferon-γ (IFN-γ), a key cytokine produced by DCs 123, promotes PANoptosis in mice. IFN-γ deficiency impaired the activation of PANoptosis-specific markers, including the CASP3, GSDMD, and MLKL, and reduced the expression of IL-1β 124. In a similar study, TNF-α and IFN-γ associated PANoptosis triggered the activation of GSDMD, GSDME, CASP3, CASP7, CASP8, and MLKL, inducing cell death in 13 distinct human cancer cell lines derived from colon and lung cancer, melanoma, and leukemia 10. This implies that PANoptosis caused by synergism of TNF-α and IFN-γ, among other immune cell cytokines, is an important mechanism to kill cancer cells and suppress tumor growth, presenting a potential therapeutic target. The participation of macrophages and DC in PANoptosis, as discussed above, is summarized in Figure 4.

### 6.3 Natural killer (NK) cells

NK cells are part of the innate immune system and play a key role in recognizing and killing cancerous and virus-infected cells. They have been shown to be involved in the induction of PANoptosis in some studies. For example, a study that analyzed the expression patterns, immunological role, and genetic alterations of PANoptosis genes in pan-cancer found that the PANoptosis pattern is associated with the infiltration of several immune cells, including NK cells 6. Similarly, a single-cell analysis of PANoptosis-related gene and tumor microenvironment infiltration in hepatocellular carcinoma revealed a PANoptosis model associated with tumor immune cell infiltration, particularly NK cells 125. In another study, the PANoptosis-associated genes, *RING finger protein 34 (RNF34)* was found to positively correlate with CD56dim NK cells and type 17 helper T cells. In contrast, *nuclear factor-kappa-B inhibitor-alpha (NFKBIA)* positively correlates with plasmacytoid DCs 126. These observations, although few, highlight the involvement of immune cells in PANoptosis; thus, detailed mechanistic studies are required to explore their therapeutic and prognostic potentials in tumors.

### 6.4 Neutrophils

**Figure 5:**
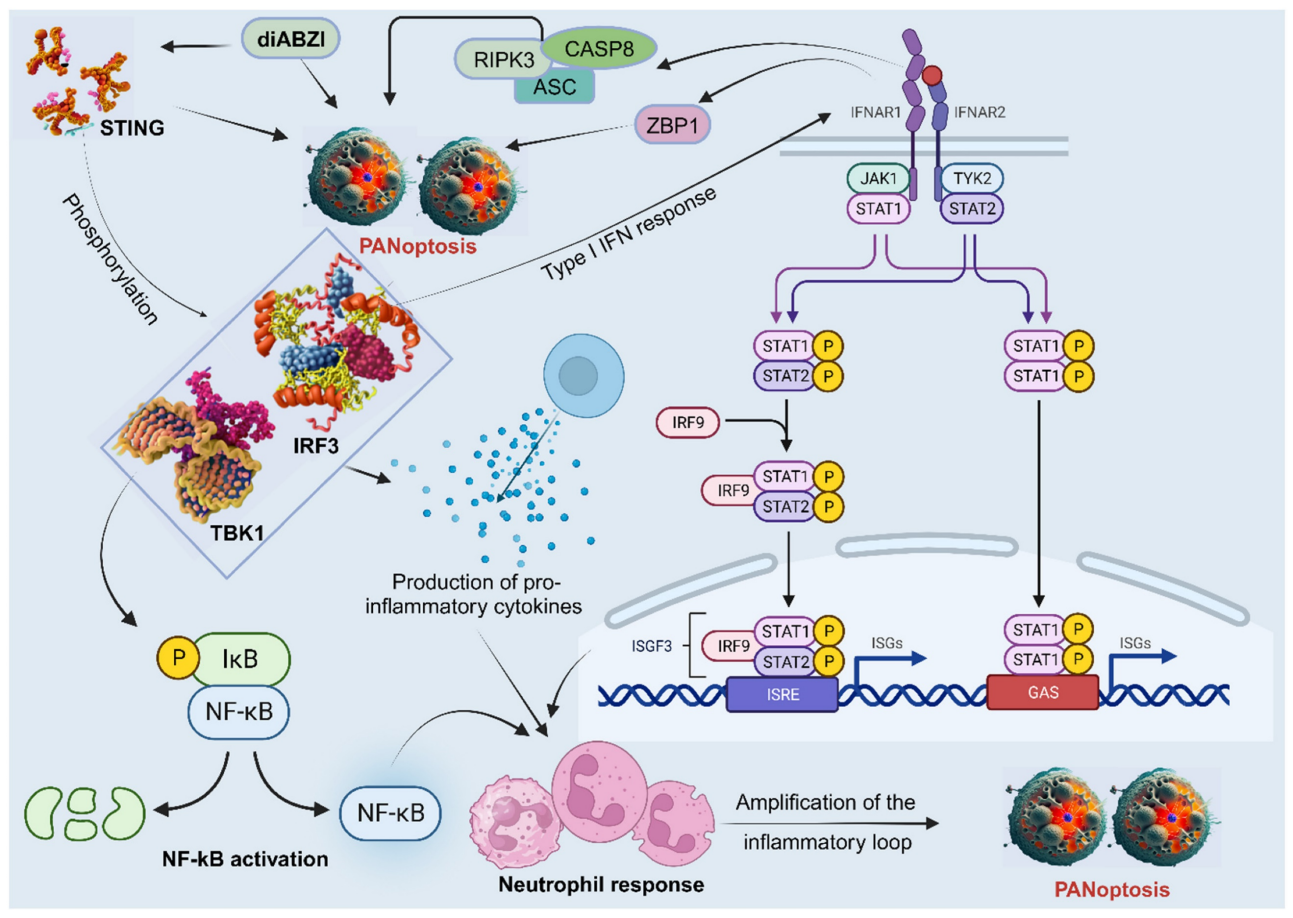
The participation of neutrophils in PANoptosis. STING agonists trigger a series of events that lead to neutrophilic inflammation and PANoptosis. First, STING activation leads to the phosphorylation of TBK1/IRF3, which triggers the production of type I interferons and NF-κB activation. This results in the production of pro-inflammatory cytokines, such as TNFα and IL-6. Next, the activation of IFNAR1 and TNFR1 signaling pathways leads to the activation of ZBP1 and RIPK3/ASC/CASP8, all involved in PANoptosis. This cascade of events, linked with neutrophil recruitment, amplifies the inflammatory response and leads to STING-dependent PANoptosis. STING, stimulator of interferon genes; TBK1, TANK-binding kinase 1; IRF, interferon regulatory factor; NF-κB, nuclear factor kappa B; IL, interleukin; TNF-α, tumor necrosis factor alpha; IFNAR1, interferon alpha/beta receptor subunit 1; TNFR1, tumor necrosis factor receptor-1; ZBP1, Z-DNA binding protein 1; CASP, caspase; ASC, apoptosis-associated speck-like protein containing a caspase recruitment domain; RIPK, receptor interacting serine/threonine kinase; JAK, Janus kinase; STAT, signal transducer and activator of transcription; IκB, IkappaB kinase; P, GAS, gamma-interferon-activated sequence; ISRE, interferon-stimulated response elements; ISGF, IFN-stimulated gene factor; ISG, IFN-stimulated gene; diABZI, diamidobenzimidazole; TYK2, tyrosine kinase 2.

Neutrophils are the most abundant type of white blood cell and play a critical role in the first-line defense against invading pathogens. They have also been shown to be involved in the induction and regulation of PANoptosis in some studies, particularly in the context of bacterial infections. The stimulator of interferon genes (STING), a PANoptosis inducer, contributes to immune responses against tumors and may control viral infection 127. A study showed that diamidobenzimidazole (diABZI), a STING agonist, induces neutrophilic response and PANoptosis. Mechanistically, the activation and dimerization of STING causes TBK1/IRF3 phosphorylation, leading to type I IFN response, NF-kB activation, and the production of pro-inflammatory cytokines TNFα and IL-6. Moreover, the activation of IFNAR1 and TNFR1 signaling pathways results in ZBP1 and RIPK3/ASC/CASP8 activation, leading to MLKL phosphorylation and necroptosis, CASP3 cleavage and apoptosis, as well as NLRP3 or AIM2 induced inflammasome formation and the subsequent release of mature IL-1β and pyroptosis 127. This cascade of events, linked with the neutrophil response, amplifies the inflammatory loop and leads to upregulation of apoptosis, pyroptosis, and necroptosis, indicative of STING-dependent PANoptosis (Figure 5) 127. However, more studies on the role of neutrophils in PANoptosis are needed.

### 6.5 Other immune cells/components

Several studies have demonstrated the involvement of other immune cells and components in PANoptosis, providing viable research areas in cancer therapy. A recent study that classified pancreatic cancer patients according to their PANoptosis-related patterns reported that patients with significantly increased infiltration of CD8^+^ T cells, monocytes, and naïve B cells had better prognosis compared to those with increased infiltration of macrophages, activated mast cells, and DCs 128. However, the mechanisms of PANoptosis in cancer related to these cells are mainly lacking. In another study that investigated the participants of PANoptosis in regulating tumor progression, the findings suggested that immune-related signaling pathways potentially mediate the role of PANoptosis-related genes in tumorigenesis. This included the enrichment of lymphocyte-mediated immunity, activation of immune response, positive regulation of leukocyte activation, positive regulation of lymphocyte activation, T cell receptor complex, immunoglobulin complex, circulating immunoglobulin complex, and immunoglobulin receptor complex 115. Similarly, it is reported that *radixin (RDX)*, a PANoptosis prognosis-related gene, is widely involved in signaling pathways such as immune response and cell proliferation in breast cancer 118. It is important to note that the involvement of these immune components and genes in PANoptosis is still an active area of research, and new findings may further expand our understanding of the specific cell types involved in this process.

## 7. Therapeutic implication of PANoptosis in cancer

### 7.1 Targeting key PANoptosis regulators

A number of studies have focused on the therapeutic target of the central modulators of PANoptosis, including ZBP1, RIPK3, MLKL, CASP8, and CASP6. ZBP1 is central in the assembly of the PANoptosome by recruiting RIPK3, which phosphorylates MLKL, leading to the translocation of MLKL to the plasma membrane to form pores that cause cell death 1,116,129. Moreover, the homo-oligomerization of MLKL is required for its translocation. According to a study, MLKL oligomerization leads to the translocation of MLKL to lipid rafts of the plasma membrane, and the plasma membrane MLKL complex acts either by itself or via other proteins to increase the sodium influx, which increases osmotic pressure, eventually leading to membrane rupture 130. CASP8 plays a role in PANoptosis by cleaving and activating CASP6, which cleaves MLKL, and leads to cell death. However, when CASP8 is inhibited, RIPK1 can form a complex with RIPK3 and FADD, which is responsible for initiating necroptosis and PANoptosis^2^. Due to the intricate role of these proteins in PCD, they form critical targets in cancer therapies that seek to regulate PANoptosis.

**Figure 6:**
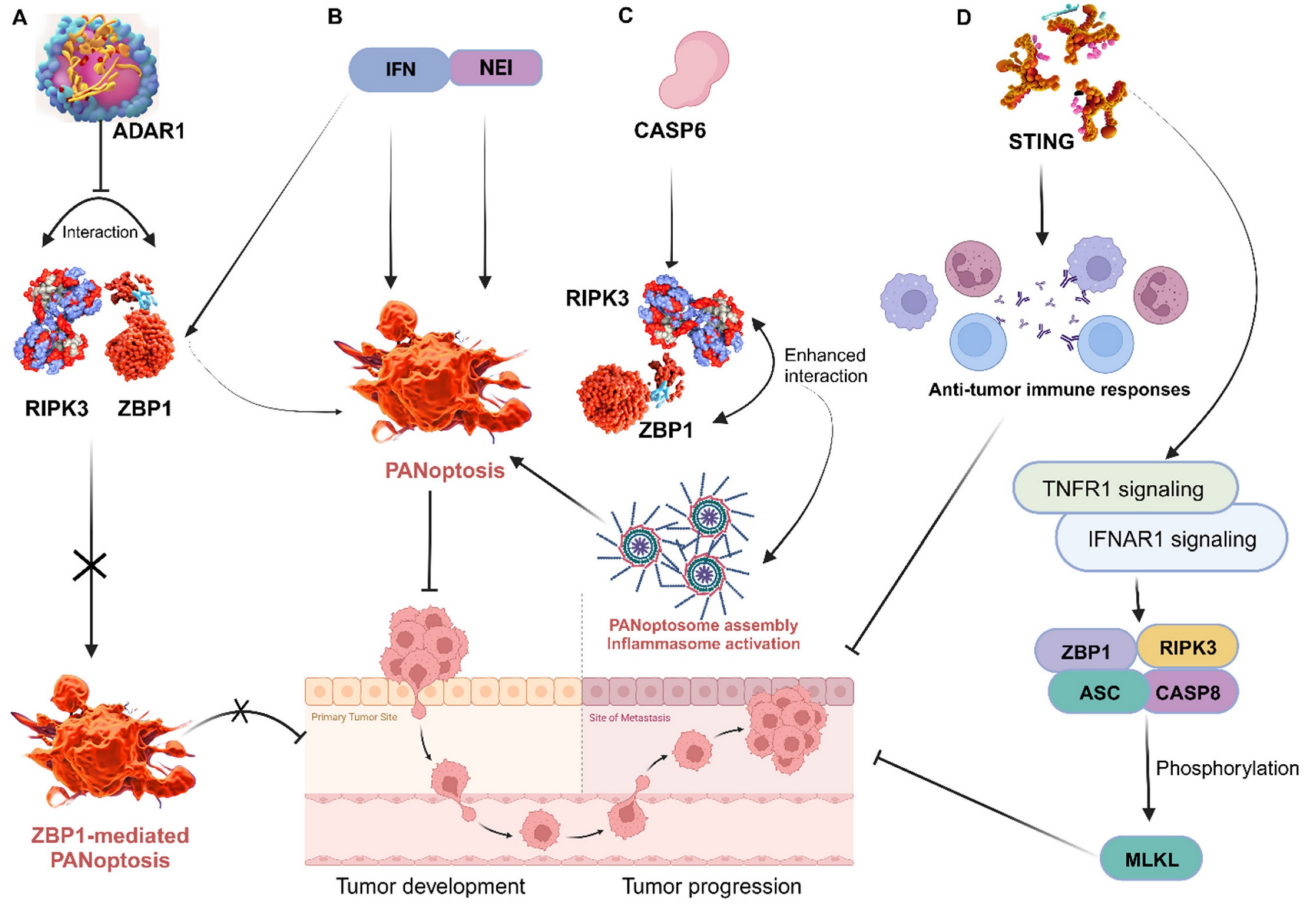
Targeting key PANoptosis regulators. A, ADAR1 impedes the interaction between ZBP1 and RIPK3, preventing ZBP1-mediated PANoptosis and promoting tumorigenesis; B, Treatment with NEI and IFN induce PANoptosis to impede tumor progression; C, The interaction of CASP6 with RIPK3 enhances that of RIPK3 and ZBP1, leading to PANoptosome assembly, which promotes ZBP1-mediated inflammasome activation and PANoptosis; D, STING enhances anti-tumor immune responses by activating TNFR1 and IFNAR1 signaling pathways, resulting in ZBP1 and RIPK3/ASC/CASP8 activation and consequently, MLKL phosphorylation and cell death. ZBP1, Z-DNA binding protein 1; ADAR1, adenosine deaminase acting on RNA 1; RIPK, receptor interacting serine/threonine kinase; NEI, nuclear export inhibitors; IFN, interferon; CASP, caspase; STING, stimulator of interferon genes; IFNAR1, interferon alpha/beta receptor subunit 1; TNFR1, tumor necrosis factor receptor-1; ASC, apoptosis-associated speck-like protein containing a caspase recruitment domain; MLKL, mixed lineage kinase domain like pseudokinase.

ZBP1 triggers inflammatory cell death and PANoptosis, whereas adenosine deaminase acting on RNA 1 (ADAR1) serves as an RNA editor that maintains homeostasis. A recent study identified and characterized ADAR1's interaction with ZBP1 to clarify its role in regulating cell death and tumorigenesis. The researchers found that ADAR1 restricts PANoptosis by interacting with the Zα2 domain of ZBP1 to suppress ZBP1 and RIPK3 interaction. Mice with a mutation in the ADAR1 gene (Adar1^fl/fl^LysM^cre^) were resistant to developing colorectal cancer and melanoma, but ZBP1 Zα2 domain deletion restored tumorigenesis in these mice. Further analysis produced a conclusion that ADAR1 inhibits ZBP1-mediated PANoptosis, leading to the promotion of tumorigenesis 110. This offers important information on the functions of ZBP1 and ADAR1, informing potential therapeutic strategies for cancer and other diseases. In other studies, treatment of murine bone marrow-derived macrophages (BMDMs) with nuclear export inhibitors (NEIs) increases the incidence of cell death by sequestering ADAR1 to the nucleus. Additionally, the use of interferons (IFNs) to induce ZBP1 also increases the incidence of cell death. Thus, emerging therapies are exploring the efficacy of the combination of NEI and IFN treatment to rapidly reduce tumor size and growth by inducing PANoptosis 105 (Figure 6). This is a highly viable area worth further exploration in cancer therapy.

Through its regulation of PANoptosis, CASP6 plays a vital role in tumorigenesis in humans and mediates anti-tumour immunity. Thus, a detailed exploration of CASP6 function in cancer via PANoptosis is important for preventing and treating tumors 108. It is reported that CASP6 interacts with RIPK3 to enhance the interaction between RIPK3 and ZBP1, thus promoting PANoptosome assembly 131 (Figure 6). This can be targeted to improve cancer cell death in treatments. In another study, CASP6 facilitated ZBP1-mediated inflammasome activation, PANoptosis cell death, and host defense, opening additional avenues for treating cancer, infectious, and autoinflammatory diseases 119. CASP 8 is another enzyme that plays a role in PCD and is activated by a variety of signals, including those from cytotoxic drugs. When CASP8 is activated, it triggers a cascade of events that leads to cell death 132. It can mediate apoptosis, pyroptosis, and necroptosis; thus, the activation or deletion of CASP8 enzyme activity is closely related to PANoptosis 133. Further exploration of the role of these caspases in tumors could lead to therapeutic and prognostic breakthroughs in cancer research. In addition, STING, a positive regulator of anti-tumor immune responses, is known to activate TNFR1 and IFNAR1 signaling pathways, resulting in ZBP1 and RIPK3/ASC/CASP8 activation and, consequently, MLKL phosphorylation and cell death 127 (Figure 5). These are viable therapeutic targets that aims at the core mechanisms associated with the execution of PANoptosis, thus require further exploration.

### 7.2 Targeting metabolic pathways and enzymes

**Figure 7:**
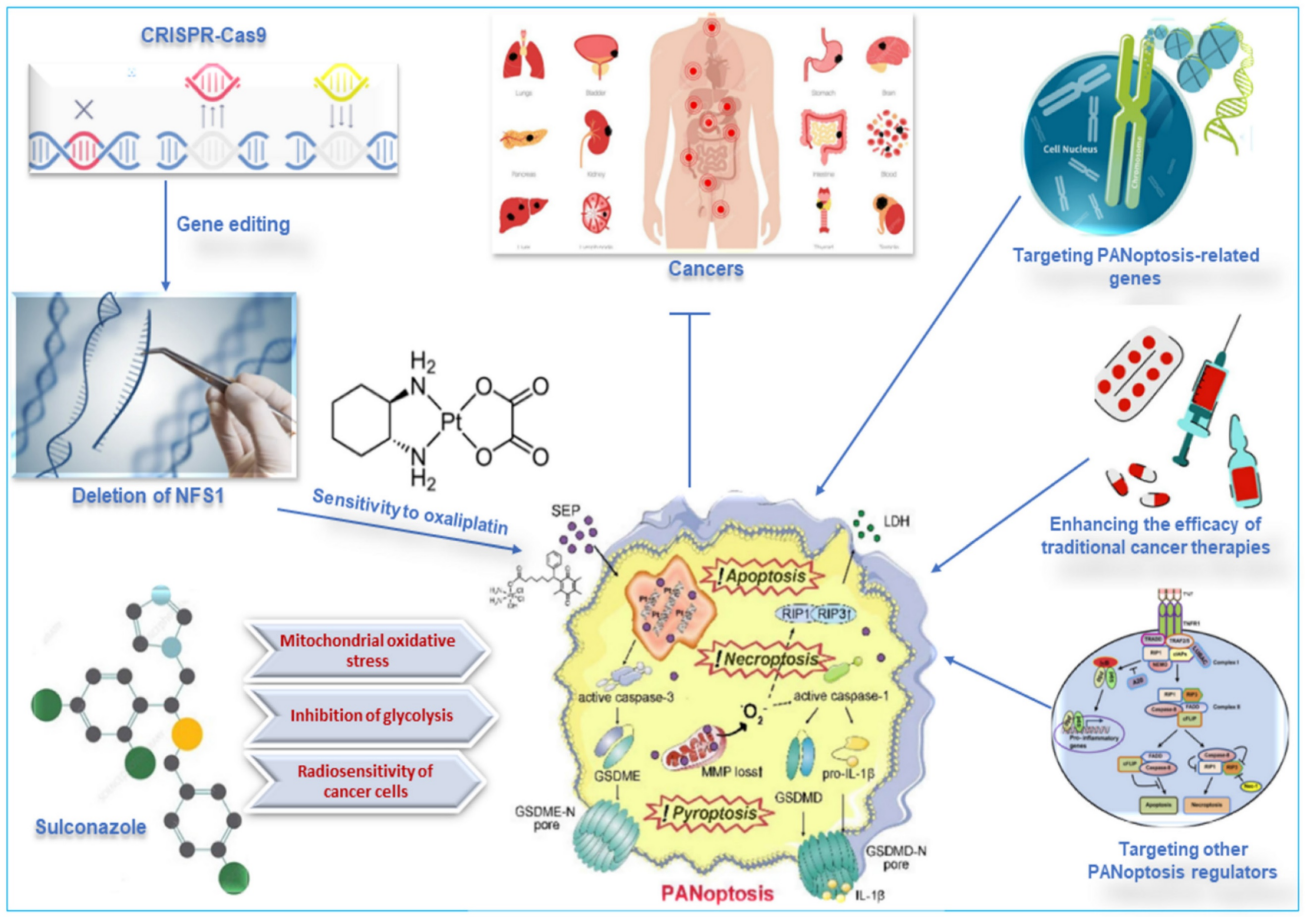
Other therapeutic targets of PANoptosis in cancer. Deleting NFS1 using CRISPR-CASP9 makes CRC cells more sensitive to the chemotherapy drug oxaliplatin by enhancing PANoptosis. Thus, oxaliplatin-triggered PANoptosis serves as a viable therapeutic target in cancer. Sulconazole inhibits cancer by inducing PANoptosis through the trigger of mitochondrial oxidative stress, inhibition of glycolysis, and elevation of the radiosensitivity of cancer cells. Other targets of PANoptosis in cancer therapy include PANoptosis-related genes, other PANoptosis regulators, and inducing PANoptosis to improve the efficacy of traditional cancer therapies. CRISPR, clustered regularly interspaced short palindromic repeats; NFS1, a gene that encodes cysteine desulfurase; CASP, caspase; CRC, colorectal cancer.

Metabolic enzymes are responsible for converting food into energy and other molecules that the body needs. Changes in the expression or activity of metabolic enzymes can affect how sensitive cells are to chemotherapy drugs. A study used CRISPR-Cas9 to delete the gene for cysteine desulfurase (NFS1), a metabolic enzyme involved in energy production, in colorectal cancer cells (CRC). The study found that deleting NFS1 made the CRC cells more sensitive to the chemotherapy drug oxaliplatin by enhancing PANoptosis. The study also found that NFS1 deficiency synergizes with oxaliplatin-triggered PANoptosis, by increasing the intracellular accumulation of ROS 12. The findings of this study suggest that NFS1 may be a potential target for developing new cancer therapies via oxaliplatin-triggered PANoptosis. The Food and Drug Administration-approved drug, sulconazole, exhibits a spectrum of anticancer effects, including the induction of various types of PCD (apoptosis, necroptosis, pyroptosis, and ferroptosis), and inhibition of proliferation and migration of cancer cells, as well as glycolysis and its related pathways. A study found that sulconazole effectively inhibits cancer by inducing PANoptosis through the trigger of mitochondrial oxidative stress and inhibition of glycolysis, as it also increases the radiosensitivity of cancer cells 134. These observations indicate that metabolic pathways such as glycolysis and metabolic enzymes such as NFS1 are crucial in not only triggering PANoptosis in cancer cells but increasing their susceptibility to radiotherapy and chemotherapy. This application, among other target routes of PANoptosis, is summarized in Figure 7.

### 7.3 Targeting PANoptosis-related genes

Resistance to PCD is a hallmark of cancer, thus, components of the cell death mechanism have long been investigated for their therapeutic, diagnostic, and prognostic potentials. A recent study that characterized PANoptosis, also a unique, innate immune-mediated inflammatory PCD pathway, reports the pan-cancer clinical significance of PANoptosis and identifies potential biomarkers. The authors found that the increased expression of PANoptosis genes *ZBP1, ADAR, CASP2, CASP3, CASP4, CASP8,* and *GSDMD* is detrimental in low-grade glioma across multiple survival models, while *AIM2, CASP3, CASP4,* and *TNFRSF10* also negatively influenced kidney renal cell carcinoma. On the other hand, the elevated expression of PANoptosis genes *ZBP1, NLRP1*(NLR family pyrin domain containing 1)*, CASP8,* and *GSDMD* was beneficial in skin cutaneous melanoma. Further therapeutic exploration indicated that treating melanoma cells via the activation of ZBP1-induced PANoptosis offers PANoptosis as a critical innate immune biomarker that can be targeted to improve patient outcomes in cancers 114. As a relatively new area, the mechanistic involvement of these genes in PANoptosis should be explored in detail, particularly toward therapeutic targets in cancer and other diseases.

### 7.4 Targeting immune components

The effectiveness of immunotherapy relies on the cytokines and cytotoxic functions of effector immune cells to bypass the resistance to cell death and eliminate cancer cells. In the context of targeting immune cells in cancer treatment, PANoptosis is activated to induce inflammatory cell death, mediated by the synergism of cytokines, such as TNF-α and IFN-γ, which are produced by immune cells 10. Mechanistically, TNF-α and IFN-γ induced PANoptosis cell death in cancer and suppress tumor growth by activating GSDMD, GSDME, CASP8, CASP3, CASP7, and MLKL 10. Specific immune cells like macrophages can secrete TNF-α and IFN-γ 10, NK cells can directly kill cancer cells by secreting perforins and granzymes and through a variety of mechanisms including PANoptosis 135, and DCs can present cancer antigens to T cells and secrete cytokines that can induce PANoptosis 10,136. These observations indicate the intricate relationship between PANoptosis and inflammasomes within innate immune cells, presenting a potential treatment strategy worth further exploration. By engineering extracellular vesicles which can induce tumor highly immunogenic PANoptosis, the researchers provided evidence that immunogenic PANoptotic cell death enables the reprogramming of immunosuppressive state and the enhancement of innate immune response by DAMPs release-promoted DC maturation and macrophage polarization (from M2 to M1 phenotype) 137. Thus, targeting PANoptotic cell death provides not only an avenue against immune escape but also a positive feedback immune activation gateway for overcoming immune resistance to intractable cancers.

Moreover, constructing a PANoptosis signature plays a significant role in tumor immunity, promoting immune cell infiltration and enhancing the overall antitumor immune response against prostate adenocarcinoma. According to the authors, PANoptosis boosts tumor-specific immunity and positively correlates with infiltration of immune cells such as CD4, CD8, and NK cells in the TME. Immune checkpoints, including CCL2, CD274, CD4, CXCR4, and LAG3, positively correlate with the PANoptosis signature 138. A robust innate immune response can significantly enhance the effectiveness of T cell-mediated anti-tumor immunity by priming, expanding, and promoting the infiltration of tumor-specific T cells into the tumor microenvironment 139. Thus, PANoptosis is significantly involved in tumor immunity as it is linked with the promotion of immune cell infiltration, increases in the expression of immune checkpoint regulators, and rise of tumor immunogenicity. Further explorations of the correlation and mechanism of these immune variables and PANoptosis patterns may provide practical ways of enhancing tumor treatment. In addition, PANoptosis-related signature indicates gliomas' prognosis and immune infiltration features 140 and predicts patient survival and immune landscape in colon cancer 7. The relationship between immune components and PANoptosis offers therapeutic and prognostic potential in cancer.

### 7.5 Enhancing the efficacy of traditional cancer therapies

In cancer, PANoptosis can be induced by a variety of factors, including chemotherapy, radiation therapy, and immunotherapy. PANoptosis can lead to the death of cancer cells, and it can also help to activate the immune system against cancer 129,141. There is growing evidence that PANoptosis may be a promising target for cancer therapy. PANoptosis-inducing agents effectively kill cancer cells in vitro and in vivo 11,142. Additionally, PANoptosis-inducing agents have been shown to enhance the efficacy of traditional cancer therapies, such as chemotherapy and radiation therapy, while some traditional cancer therapies could also induce PANoptosis. For example, a recent study identified FADD, an adaptor of PANoptosis and apoptosis, as a prominent risk factor in lung cancer, mainly localized in nucleoplasm and cytosol. Biological enrichment and immune infiltration analyses show that patients with a high expression of FADD respond better to chemotherapies such as 5-Aminoimidazole-4-carboxamide ribonucleotide (AICAR), bortezomib, docetaxel, and gemcitabine compared to immunotherapy 143. However, in another study, the inhibition of FADD significantly reduced the ability of lung cancer cells to proliferate, and its knockdown promoted apoptosis and pyroptosis, presenting this PANoptosis-related gene as a potential treatment target in cancer 143. Regardless, the effect of FADD may vary between cancer therapies and require in-depth exploration. A similar study also reported the significant value of the PANoptosis pattern in serving as a predictive indicator of immunotherapy response in patients with tumors 6.

PANoptosis has emerged as a promising strategy for enhancing immunotherapy efficacy. By inducing a combination of apoptosis, pyroptosis, and necroptosis, PANoptosis can effectively eliminate cancer cells while eliciting a robust anti-tumor immune response 144. Several mechanisms contribute to the synergistic effects of PANoptosis and immunotherapy, including enhanced tumor antigen release, increased expression of immune checkpoint molecules, and reduced tumor immunosuppressive environment. For instance, PANoptosis has been shown to upregulate the expression of immune checkpoint molecules, such as PD-L1, on cancer cells 140. These molecules can be targeted by immune checkpoint inhibitors (ICIs), which can further enhance the anti-tumor activity of T cells 140,144. PANoptosis can also disrupt the immunosuppressive environment that often surrounds tumors by eliminating immunosuppressive cells, such as Tregs, and reducing the production of immunosuppressive factors, such as TGF-β 144. Further research is needed to elucidate the mechanisms underlying these effects and to develop novel therapeutic strategies that combine PANoptosis-inducing agents with immunotherapy.

### 7.6 Targeting other PANoptosis regulators

By analyzing the adrenocortical carcinoma dataset in the cancer genome atlas (TCGA), a study found that the increased expression of cyclin-dependent kinase-1 (CDK1) is significantly associated with the adverse clinical outcomes of adrenocortical carcinoma. Further in vitro and in vivo experiments indicated that the overexpression of CDK1 promotes proliferation and epithelial-to-mesenchymal transition (EMT), partly by regulating PANoptosis of adrenocortical carcinoma cells through binding with the PANoptosome in a ZBP1-dependent way 142. The subsequent target of CDK1 via inhibitors such as cucurbitacin E (CurE), with and without mitotane exhibited good safety and efficacy for the treatment of cancer 142. NLRP12 initiates inflammasome and PANoptosome activation, resulting in cell death and inflammation upon exposure to heme/PAMPs or TNF. The induction of NLRP12 expression through TLR2/4-mediated signaling via IRF1 leads to inflammasome assembly, triggering the maturation of IL-1β and IL-18. This inflammasome, integral to a larger NLRP12-PANoptosome, orchestrates inflammatory cell death through the involvement of CASP8/RIPK3 145. This identifies NLRP12 as an essential cytosolic sensor for heme/PAMPs-mediated PANoptosis and inflammation, suggesting that NLRP12 and its pathway-associated molecules are potential drug targets in the induction of PANoptosis.

Interferon regulatory factor 1 (IRF1) is a protein that regulates many different biological processes, including the response to cancer. Mutations in the IRF1 gene or changes in its function have been linked to several types of cancer 146,147. IRF1 plays a role in controlling cell growth, differentiation, and death. It also helps to regulate the immune system. When IRF1 is not working properly, it can lead to uncontrolled cell growth and division, which can eventually lead to cancer 146,148. A study demonstrated that the lack of IRF1 results in hyper-susceptibility of colorectal tumorigenesis. Further analysis indicated that IRF1 protects against both AOM/DSS-induced and spontaneous colorectal cancer in mice, where the attenuated cell death in mice colon was due to defective PANoptosis (apoptosis, pyroptosis, and necroptosis) 13. This identifies IRF1 as an upstream regulator of PANoptosis to induce cell death, serving as a potential therapeutic target in cancer. Table 2 summarizes targets of PANoptosis in recent studies in cancer.

### 7.7 The evolving application of PANoptosis in nanomedicine

In nanomedicine, nanoparticles such as extracellular vesicles, liposomes, dendrimers, and polymers are used to deliver drugs, genes, or other therapeutic agents to cells. Nanoparticles have been shown to be effective in inducing PANoptosis in cancer cells 150. A recent study reports an ultrasound nanomedicine-triggered tumor immune-reediting therapeutic strategy that employs nano/genetically engineered extracellular vesicles. These vesicles have the potential to trigger a highly immunogenic form of PANoptosis cell death within tumors. This process iteratively initiates the activation of the cancer's innate immunity cycle by releasing DAMPs multiple times. Consequently, it primes many antigen-specific T cells and shapes a protective immune response by activating the cGAS-STING signaling pathways 137. With the aid of immune checkpoint blockade, reprogramming the immune microenvironment further enhanced a prompt bridging of innate and adaptive immunity and significantly inhibited metastatic and rechallenged tumor growth. Moreover, intra-tumor levels of specific critical cytokines, including IL-2, TNF-α, IFN-γ, and IL-6, which are secreted by immune cells for promoting T-cell responses, were remarkably elevated along with PANoptosis-augmented recruitment of CD8^+^ and CD4^+^ T cells in tumor tissues 137. These observations indicate that targeting PANoptosis cell death via nanoengineering may offer unique opportunities to boost anti-tumor immunological effects.

In addition, PANoptosis can be induced by various nanoparticles, including gold, silver, and carbon nanotubes by activating different signaling pathways, such as the RIPK1-RIPK3 axis, the CASP8-CASP3 axis, and the MLKL pathway, effectively eliminating cancer cells, suppressing tumor growth, and enhancing traditional cancer therapies 2,141. Overall, PANoptosis is a promising new target in nanomedicine for cancer therapy, although it is still in its early stages of development. Further research is needed to develop more effective and safer nanoparticle-based treatments for cancer.

## 8. Conclusion and perspective

As a recently described form of cell death, PANoptosis is characterized by the simultaneous execution of apoptosis, necrosis, and pyroptosis. It is a complex process regulated by various signaling pathways, and it is implicated in a wide range of diseases, including cancer, infection, and inflammation. The available literature on PANoptosis is still in its early stages, but it is rapidly expanding with promising initial findings that suggest that it could be a valuable therapeutic target. The prospects of research on PANoptosis in cancer are up-and-coming, as PANoptosis has been shown to participate in the development and progression of cancer and is involved in resistance to cancer treatment. This indicates that PANoptosis could be a new and effective target for cancer therapy.

Some of the specific areas of future research on PANoptosis in cancer that have the potential to lead to new therapeutic advances include identifying the molecular mechanisms of PANoptosis in cancer. A better understanding of the molecular mechanisms of PANoptosis in cancer could lead to the development of new drugs that can specifically target this process. Moreover, developing new drugs to induce PANoptosis in cancer cells is a promising approach since such drugs could kill cancer cells and shrink tumors. On the other hand, drugs that inhibit PANoptosis in cancer cells could be used to overcome resistance to cancer treatment and improve the effectiveness of existing therapies. Furthermore, future research could also explore developing new drugs that target the tumor microenvironment to induce PANoptosis. Since the tumor microenvironment can protect cancer cells from PANoptosis, therapies that can target the tumor microenvironment to induce PANoptosis could be used to make cancer cells more susceptible to PANoptosis-inducing therapies. In addition to these specific areas of research, a number of general challenges need to be addressed to develop new PANoptosis-based cancer therapies. For example, developing drugs that can specifically target PANoptosis in cancer cells without harming healthy cells is important. It is also important to develop biomarkers that can be used to identify patients who are likely to respond to PANoptosis-based therapies.

In conclusion, the prospects of research on PANoptosis in cancer are very promising. PANoptosis is a new and exciting area of cancer research, and there is a great deal of potential to develop new and effective PANoptosis-based cancer therapies.

## Figures and Tables

**Table 1:** Evidence of PANoptosis in cancer

Type of cancer	Study type	Analytical technique	Key finding	Reference
Lower-grade glioma	Clinical/patient samples	Database analysis of 1203 lower-grade glioma samples	Distinct PANclusters have their own molecular, clinical, and immunological profiles, and PANoptosis pattern predicts clinical survival	113
Colon adenocarcinoma	Clinical/patient samples, cell culture	Bioinformatics analysis of TCGA and GEO databases	lncRNAs are associated with metastasis and PANoptosis. lncRNA SNHG7 is linked with tumor stage and lymph node metastasis	111
Gastric and pan-cancer	Clinical/patient samples	Data acquisition and processing of RNA-seq data and corresponding clinicopathological information of samples from different databases	PANoptosis pattern score is linked with the response rate to immunotherapy and prognosis	3
Pan-cancer	Clinical/patient samples	A bioinformatic approach using the Human Protein Atlas database and RT-PCR	PANoptosis genes and scores are significantly linked with patient survival, immune and inflammatory responses, and tumor microenvironment	6
Colon	Clinical/patient samples	Bioinformatics analysis of PANoptosis-related genes and clinical data from TCGA and GEO databases	PANoptosis-related genes and gene clusters correlate with patient survival, immune system, and cancer-related biological processes and pathways	7
Cutaneous melanoma	Clinical/patient samples	Data collection and processing from TCGA	PANoptosis-related genes (*ZBP1, MAP3K7, and RBCK1*) could be used for risk assessment and OS prediction, reflect the immune landscape of patients and provide a novel reference for individualized tumor treatment	115
Breast	In vitro cell line and clinical data access.	Breast cancer and GSE176078 single-cell sequencing dataset from GEO and TCGA databases	PANoptosis pattern in a low-risk group correlates with better prognosis and higher levels of immune infiltration and immune checkpoint-related genes	118

RT-PCR, real-time quantitative reverse transcription polymerase chain reaction; TCGA, the cancer genome atlas; GEO, gene expression omnibus; OS, overall survival; ZBP1, Z-DNA binding protein 1; MAP3K7, mitogen-activated protein kinase kinase kinase 7.

**Table 2:** The potential targets of PANoptosis in cancer therapies

Type of cancer	Study model	Therapy/target molecule	Key finding	Reference
Colorectal	Clinical samples, in vivo (ALB/c nude mice), and in vitro cells	Treatment with Oxaliplatin	NFS1 deficiency synergizing with oxaliplatin triggered PANoptosis	12
Colorectal	In vivo (Irf1-/- mice) of AOM/DSS-induced CRC, a spontaneous mouse model of CRC, and in vitro cells	Defective PANoptosis	IRF1 attenuates cell death in the colons of mice; lack of IRF1 induces hypersusceptible to colorectal cancer	13 149
Melanoma and colorectal	In vivo *mice (Adar1^fl/fl^LysM^cre^Zbp1^-/-^ and Adar1^fl/fl^LysM^cre^Zbp1^ΔZα2^)*, and in vitro cells	Targeting ADAR1, ZBP1	ADAR1 suppresses ZBP1-mediated PANoptosis to promote tumorigenesis	110
Kidney renal cell carcinoma, low-grade glioma, and skin-cutaneous melanoma	Clinical sample data acquisition in databases, in vitro cells	Treatment with KPT-335 or LMB with or without IFN-γ/ Targeting PANoptosis genes, e.g., ZBP1 in melanoma cells	*AIM2, CASP3, CASP4,* and *TNFRSF10* negatively affect renal cell carcinoma. *ZBP1, ADAR, CASP2, CASP3, CASP4, CASP8,* and *GSDMD* negatively affect low-grade glioma. *ZBP1, NLRP1, CASP8,* and *GSDMD* positively affect skin cutaneous melanoma	114
Adrenocortical carcinoma	In vitro (NCI-H295R and SW-13 cells), in vivo (female BALB/c-nu nude mice), and TCGA database	Treatment with cucurbitacin E with or without mitotane	Regulates EMT, the G2/M checkpoint, and PANoptosis. Exhibits good safety and efficacy in the treatment	142
Lung	Clinical samples data acquisition in databases (GEO and TCGA), in vitro (BEAS-2B, A549, H1299, and H441 cells)	Chemotherapies (AICAR, bortezomib, docetaxel, and gemcitabine) and immunotherapy/targeting FADD	Increased levels of FAAD positively correlate with response to the chemotherapies but negatively with immunotherapy. Inhibition of FADD prevents tumor cell proliferation, and knockdown promotes apoptosis and pyroptosis.	143
Esophageal	In vitro cell lines	Treatment with sulconazole	Induces PANoptosis by triggering oxidative stress and inhibiting glycolysis to increase radiosensitivity in cancer. Inhibits the proliferation and migration of cancer cells	134

AIM2, absent in melanoma 2; CASP, caspase; NFS1, cysteine desulfurase; ZBP1, Z-DNA binding protein 1; ADAR1, adenosine deaminase acting on RNA 1; LMB, leptomycin B; TCGA, the cancer genome atlas; FADD, Fas-associated death domain; AICAR, 5-Aminoimidazole-4-carboxamide ribonucleotide; NLRP1, NLR family pyrin domain containing 1; GSDMD, Gasdermin D.
